# Iron Dysregulation in Human Cancer: Altered Metabolism, Biomarkers for Diagnosis, Prognosis, Monitoring and Rationale for Therapy

**DOI:** 10.3390/cancers12123524

**Published:** 2020-11-26

**Authors:** Pierre Lelièvre, Lucie Sancey, Jean-Luc Coll, Aurélien Deniaud, Benoit Busser

**Affiliations:** 1 Institute for Advanced Biosciences, UGA INSERM U1209 CNRS UMR5309, 38700 La Tronche, France; pierre.lelievre@univ-grenoble-alpes.fr (P.L.); lucie.sancey@univ-grenoble-alpes.fr (L.S.); jean-luc.coll@univ-grenoble-alpes.fr (J.-L.C.); 2 Univ. Grenoble Alpes, CNRS, CEA, IRIG, Laboratoire de Chimie et Biologie des Métaux, 38000 Grenoble, France; 3 Department of Clinical biochemistry, Grenoble Alpes University Hospital, 38043 Grenoble, France

**Keywords:** iron homeostasis, cancer, prognostic, diagnostic, therapy

## Abstract

**Simple Summary:**

Iron is the more abundant metal ion in humans. It is essential for life as it has a role in various cellular processes involved, for instance, in cell metabolism and DNA synthesis. These functions are crucial for cell proliferation, and it is therefore not surprising that iron is accumulated in tumors. In this review, we describe normal and altered iron homeostasis mechanisms. We also provide a vision of iron-related proteins with altered expression in cancers and discuss their potential as diagnostic and/or prognostic biomarkers. Finally, we give an overview of therapeutic strategies acting on iron metabolism to fight against cancers.

**Abstract:**

Iron (Fe) is a trace element that plays essential roles in various biological processes such as DNA synthesis and repair, as well as cellular energy production and oxygen transport, and it is currently widely recognized that iron homeostasis is dysregulated in many cancers. Indeed, several iron homeostasis proteins may be responsible for malignant tumor initiation, proliferation, and for the metastatic spread of tumors. A large number of studies demonstrated the potential clinical value of utilizing these deregulated proteins as prognostic and/or predictive biomarkers of malignancy and/or response to anticancer treatments. Additionally, the iron present in cancer cells and the importance of iron in ferroptosis cell death signaling pathways prompted the development of therapeutic strategies against advanced stage or resistant cancers. In this review, we select relevant and promising studies in the field of iron metabolism in cancer research and clinical oncology. Besides this, we discuss some co-existing discrepant findings. We also present and discuss the latest lines of research related to targeting iron, or its regulatory pathways, as potential promising anticancer strategies for human therapy. Iron chelators, such as deferoxamine or iron-oxide-based nanoparticles, which are already tested in clinical trials, alone or in combination with chemotherapy, are also reported.

## 1. Introduction

Iron (Fe) is one of the most important trace elements for eukaryotic cells, with countless cellular roles. It is a co-factor of many ferro-dependent enzymes such as the enzyme involved in DNA synthesis and repair, as well as cellular energy production and oxygen transport. It is also present in many hemoproteins, such as hemoglobin or myoglobin. Functional Fe protects cells from the formation of free radicals through its involvement in catalases and peroxidases. Iron cellular homeostasis is highly regulated because both deficiency and excess of Fe have deleterious cellular effects.

In cancer cells, these biological processes are also central for the acquisition of malignant phenotypes, and the dysregulation of Fe-related proteins actively participates in oncogenesis. Some studies have also revealed that these dysregulations could be of clinical interest as prognostic and/or predictive biomarkers of response to treatment. Accordingly, several therapeutic strategies targeting or using trace elements have been developed. In view of this rich literature, we present some of the most significant studies with results and cell mechanisms relating to Fe homeostasis dysregulation and cancer. This review is also an opportunity to present the discrepant results on this subject. Finally, in this work, we review the main therapeutic strategies targeting Fe or using Fe as a central player for cancer treatment.

Numerous studies have shown that Fe contributes to carcinogenesis and metastatic processes. Altered Fe metabolism and cancer patients’ prognoses are linked. The use of these biomarkers could therefore contribute to clinical decision-making. Finally, understanding the dysregulation of iron metabolism in tumors allows better patient care as well as the implementation of new therapeutic strategies.

## 2. Iron Normal Metabolism

Iron is a trace element essential for mammals. This trace element is determinant for the transport of oxygen in the blood as well as energy production in the mitochondria, muscle function, and hematopoiesis [1,2]. In addition, Fe is a co-factor for many enzymes involved in mitosis or in detoxifying mechanisms, for instance. The systemic Fe homeostasis is mainly maintained through the recycling of senescent erythrocytes by macrophages and Fe is stored in hepatocytes. These mechanisms contribute to 90% of the needs, the remaining being absorbed from the diet to counterbalance iron losses [3]. Three to four grams of Fe are present in the human body, while Fe plasma concentration is between 10 and 30 µM, so that the iron in plasma represents only around 6 mg of iron on average, a tiny percentage of total iron. Dysregulation of iron homeostasis provokes either cellular dysfunction, leading to anemia if there is a negative Fe regulation, or to tissue injury in the case of positive Fe regulation [4]. These damages come from the capacity of iron to undergo cyclic oxidation and reduction. The redox activity of Fe generates free radicals and other oxidizing species through a variety of mechanisms such as the Fenton reaction. This reaction rapidly changes free Fe in the form of Fe^3+^ to Fe^2+^ by a reduction reaction with hydrogen peroxide. In addition, this reaction leads to the production of hydroxyl radicals that can cause biological damage [5,6]. Iron is an essential trace element that can be toxic for cells and organisms; consequently, several mechanisms are implemented to regulate precisely Fe absorption, transport, and storage.

**Figure 1:**
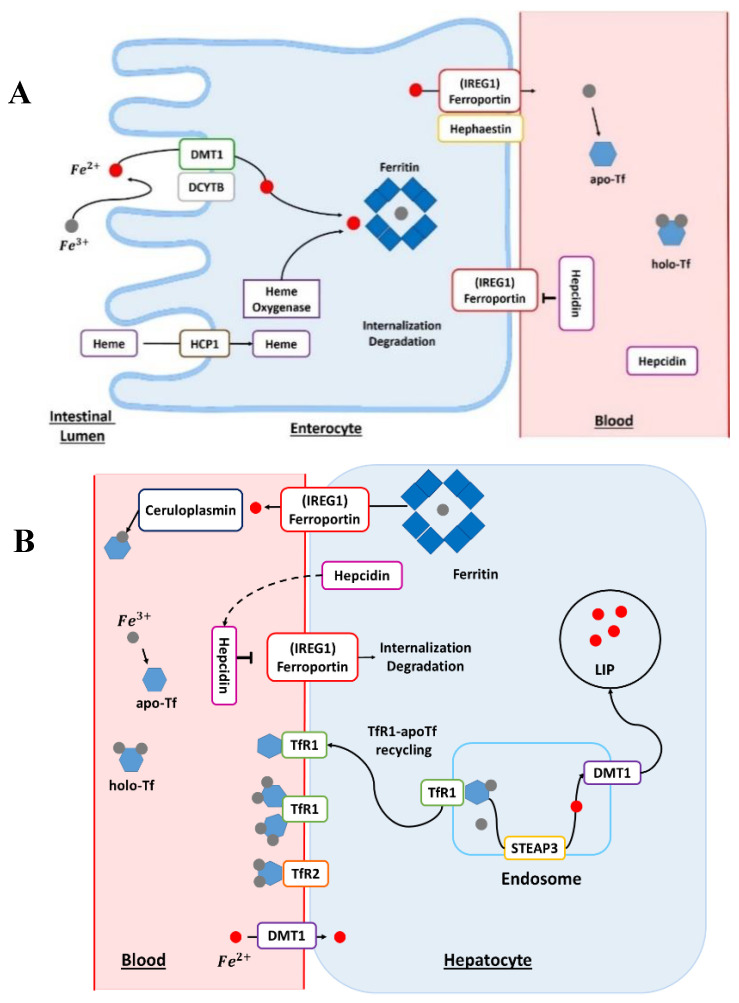
(**A**)**:** The enterocyte: absorption site of dietary heme and non-heme iron. The Fe in the diet is mainly in the form of ferric Fe (Fe^3+^). Before its absorption into the enterocyte, Fe is reduced by the action of a reductase, such as duodenal cytochrome b (DCYTB). The ferrous Fe (Fe^2+^) subsequently enters the cell via the divalent metal transporter 1 (DMT1). Heme Fe is absorbed by the action of the heme carrier protein 1 (HCP1). The heme is degraded by the action of heme oxygenase and then ferrous Fe is released. The Fe contained in the cell may be stored in ferritin-bound form or it may be delivered to the circulation by the action of ferroportin, also known as iron-regulated transporter 1 (*IREG1*). Before joining the systemic circulation, the Fe is oxidized by hephaestin; then, Fe binds to transferrin (Tf), which can bind two ferric atoms (Fe^3+^). apo-Tf, apotransferrin; holo-Tf, holotransferrin. (**B**)**:** The hepatocytes: principal storage site of iron. In blood, transferrin-bound Fe binds to transferrin receptor 1 (Tfr1) at the plasma membrane. The transferrin receptor 2 (Tfr2) protein plays the role of an Fe sensor and contributes to Fe homeostasis. For the release of Fe into the cell, the complex transferrin–Fe and Tfr1 are endocytosed. In the endosome, ferric Fe is released from transferrin (Tf) and reduced to ferrous Fe (Fe^2+^) via the six-transmembrane epithelial antigen of prostate 3 (STEAP3) protein. The transferrin–Tfr1 complex joins the plasma membrane and transferrin can participate in further cycles of Fe absorption. The Fe^2+^ is transported out of the endosome by DMT1. This Fe is part of the active labile Fe pool (LIP) and participates in cellular metabolism. In the cell, Fe can also be stored in the ferritin. Iron can exit hepatocytes via ferroportin, also known as iron-regulated transporter 1 (*IREG1*). In blood, Fe^2+^ is reoxidized by plasma ferroxidase, known as ceruloplasmin, to allow loading onto the Tf. Ceruloplasmin is a copper-dependent ferroxidase, a major protein of copper homeostasis. Hepatocytes are regulators of Fe homeostasis via the secretion of the peptide hormone hepcidin. High levels of Fe cause the production and secretion of hepcidin in the blood. Hepcidin binds to ferroportin and this triggers its degradation. apo-Tf, apotransferrin; holo-Tf, holotransferrin.

Humans absorb Fe either complexed with heme or under free form. This assimilation predominantly occurs in the small intestine. Furthermore, Fe absorption is higher in the duodenum and jejunum, with a continuous decrease from proximal to distal [7]. The reduction of Fe^3+^ into Fe^2+^ is a mandatory first step for Fe absorption by duodenal enterocytes (Figure 1A). This reduction reaction is triggered by the duodenal cytochrome b (DCYTB) transmembrane ferric reductase and mediated by intracellular ascorbate. The DCYTB could have an important role in iron homeostasis owing to various factors affecting gene regulation. After the reduction step, Fe enters the duodenal enterocyte by divalent metal transporter 1 (DMT1) [2]. The transport of iron by DMT1 is proton-coupled and thus requires a pH gradient.

The DMT1 mRNA contains an iron responsive element (IRE) in its 3′-untranslated region (UTR) [8]. This IRE/IRP (iron regulatory protein) system regulates the translation of mRNAs containing an IRE pattern. Importantly, the IRE motif is present in most of the mRNA encoding for proteins involved in Fe and energy metabolisms. In this system, the activity of IRP1 and IRP2 is regulated by independent post-transcriptional mechanisms controlled by cellular Fe levels [9]. According to many studies, the DMT1 transporter is an essential regulator of duodenal Fe uptake. The genetic knockout of DMT1 has shown that it is a fundamental element for human life and that DMT1 mutations induce defective intestinal Fe use, resulting in severe microcytic anemia at birth [10].

The absorption mechanisms of heme iron remain unclear. However, a certain number of heme transport proteins have been identified within the enterocytes, such as proton-coupled folate transporter/heme carrier protein 1 (PCFT/HCP1), cellular receptor 1 (FLVCR1), heme responsive gene 1 (HRG-1), and finally the breast cancer resistance protein (ABCG2). In addition, hemes present in the blood can be absorbed by multiple cell types, such as erythroid cells or hepatocytes for producing hemoproteins. Heme iron from the diet enters the enterocytes via the HCP1 protein. The intracellular heme is subsequently degraded by the action of heme oxygenase (Figure 1A). Iron released from the heme will join the absorbed Fe pool as inorganic non-heme Fe [11]. Iron will reach the bloodstream via the membrane protein ferroportin (IREG1), which is the only export protein currently known for non-heme Fe (Figure 1A) [12]. IREG1 is thus an essential component of systemic Fe homeostasis [13], with an IRE domain in its mRNA at the 5’-UTR level [14] and this protein has many levels of regulation. The ferroportin directly interacts with the hormone peptide hepcidin (Figure 1A). Hepcidin has a regulatory action on ferroportin through internalization and degradation of this membrane protein [4]. In addition, hepcidin is the hormone regulating Fe homeostasis at the level of the organism. Hepcidin is secreted by the liver, and its regulation can be modulated by different conditions such as anemia, inflammation, or conditions such as Fe levels in the liver and transferrin saturation [15].

The membrane protein hephaestin and the plasma protein ceruloplasmin (Cp) oxidize cellular Fe^2+^ into Fe^3+^ [2]. Ceruloplasmin is also a copper protein [16]. In the bloodstream, Fe could be bound to transferrin (Tf) for its delivery to different cell types (Figure 1A,B). Fe^3+^ is charged onto apotransferrin (Apo-Tf) to form the Fe-associated holotransferrin (holo-Tf). The holo-Tf is captured by transferrin receptors (TfR1). Transferrin is mainly produced in the liver and is 30–35% iron-saturated in physiological conditions. Transferrin can bind to different metals but it has a higher affinity for Fe^3+^ and does not bind Fe^2+^. Moreover, Fe^3+^ binding is reversible and pH-dependent [2].

The absorption of iron-bound transferrin by the cells occurs through Tfr1 and Tfr2 receptors (Figure 1B). Tfr1 is located at the surface of proliferating cells because Fe is a central element for the cell cycle, while Tfr2 receptors are expressed on hepatocytes. These receptors are the main pathway for internalizing Fe in different cells [17]. The endocytosis of the Tfr–Tf complexes results in the intra-endolysosomal release of Fe upon endosome acidification [17]. The released ferric iron is reduced in the endosome to the ferrous form by members of the metalloreductase family STEAP, the protein STEAP3 (six-transmembrane epithelial antigen of prostate protein family, member 3) [18]. The members of the STEAP family differ in their tissue expression profiles but all STEAP proteins are localized to the plasma membrane and/or endosomes. Nevertheless, STEAP3 is the main intracellular ferrireductase identified [19,20]. It was discovered that STEAP2, STEAP3, and STEAP4 have not only a ferroreductase function but also a cupric reductase activity that increases the absorption of cellular iron and copper [19]. In addition to playing a central role in Fe metabolism, STEAP proteins are also involved in the cellular regulation of copper (Cu). Ferrous iron leaves the endosome through the action of DMT1 and can be stored in a ferritin-bound form in both hepatocytes and enterocytes (Figure 1A,B), once oxidized by the ferritin’s H subunit [2]. Ferritin is an iron-binding protein that is highly conserved through evolution and whose primary function is the sequestration of iron. This protein also has a ferroxidase function, allowing the conversion of Fe^2+^ into Fe^3+^ during its internalization and sequestration. Ferritin has two subunits, H and L, in the cytosol, which assemble to form apoferritin. In addition, the ratio between these two subunits can vary considerably according to many parameters such as tissue type or cellular conditions. Besides this, many factors, such as cytokines and oncogenes, in addition to proteins regulating iron homeostasis, regulate ferritin [21]. The receptor–transferrin complexes are recycled at the surface of the hepatocyte [18]. Once in the bloodstream, Fe reaches the liver, which is the main storage site for iron. Finally, the regulation of iron homeostasis is also controlled by another mechanism: miRNAs which participate in the silencing of certain RNA and have a direct effect on the post-transcriptional regulation of the genes involved in the regulation of iron metabolism [22,23].

## 3. Iron and Fe Proteins as Cancer Biomarkers

Iron plays an important role in many processes in cancerogenesis. However, iron can also be an important mediator of cell death via ferropotosis, which is a form of programmed cell death [24]. Therefore, iron may have a tumor suppressor action. Indeed, iron’s ability to alternate between oxidized and reduced forms contributes to the formation of free radicals that will accelerate tumor initiation [25]. Ferroptosis is a cell death pathway that appears to occur as a result of two processes affecting the cell: the disruption of the cell’s antioxidant capacity and the increase in the amount of intracellular iron [24]. Moreover, iron plays a role in metastasis formation and angiogenesis via iron metalloproteases. Iron possesses the ability to modulate the microenvironment via iron matrix degradation and cancer metastasis [26,27]. Finally, iron can also promote the proliferation of tumor cells. Indeed, cancer cells have higher iron requirements due to higher rates of proliferation and DNA synthesis [28]. Consequently, remodeling of the iron metabolism pathways in cancer cells has been observed. Iron could also contribute to the progression of cancers through changes at the gene and at the epigenetic level. Actually, cancers are both genetic and epigenetic diseases, and iron participates in the regulation of both the genome and epigenome. Effectively, and as seen previously, iron regulates the transcription of various proteins directly or indirectly related to iron homeostasis via the IRE/IRP system. It should also be noted that, in addition to the IRE/IRP system, iron is involved in epigenetic modulation via Fe-S clusters. Indeed, Fe-S clusters are essential for the formation of stable and active complexes such as DNA polymerases and enzymes involved in processes of DNA repair [28]. Furthermore, it has been shown that Fe-S aggregates are also essential in the modulation of histone and tubulin acetylation [29]. Therefore, the alteration of Fe-S clusters’ biogenesis and/or iron homeostasis in cancers promote modifications at the genome and the epigenome levels [30,31].

Iron is involved in several mechanisms frequently altered in cancer cells, such as tumor cell survival or reprogramming of the tumor microenvironment. The loss of Fe homeostasis can occur at different stages of carcinogenesis, i.e., tumor initiation, progression, and metastasis. In cancer, some mRNA or protein dysregulations of key Fe metabolic players have been reported [32]. These variations could be relevant diagnostic or prognostic biomarkers in cancer (Table 1). Consequently, their use as routine clinical tests could upgrade the current medical management of cancer, ultimately leading to improvements in patients’ care.

Hepcidin is an essential hormone for the regulation of Fe efflux and it contributes to the proliferation of cancer cells. Moreover, the expression and regulation of this hormone are variable within cancer tissues [114]. The concentration of hepcidin increases in many cancers such as myeloma, Hodgkin’s disease, breast, prostate, thyroid, and non-small-cell lung cancers (NSCLC), but also in other solid tumors [33,40,82,103,106,115]. Contrariwise, hepcidin concentrations are decreased in brain cancers, hepatocellular and renal cell carcinoma, and hepatocellular carcinoma [58,62,94,114]. Numerous studies have shown that the high expression of the hepcidin mRNA predicts poor prognosis and is associated with a metastatic profile [115].

The epigenetic regulation of hepcidin actively contributes to Fe dysregulation in cancers. In a DNA-methylation-dependent mechanism, the epigenetic silencing of SOSTDC1 (a protein controlling hepcidin synthesis) induced prostate cancer progression [33]. Another epigenetic regulation of the E4BP4/G9a/SOSTDC1/hepcidin pathway induced the repression of hepcidin and thyroid cancer proliferation [106].

Iron homeostasis and inflammation are tightly linked. In the serum of multiple myeloma patients with inflammation (patients with plasma C-reactive protein > 10 mg/dL), hepcidin is abnormally increased, together with interleukin-6 (IL-6) [116,117]. IL-6 is a cytokine involved in the acute phase of inflammation, which stimulates the production of hepcidin via a transcriptional control depending on STAT3 interactions. This control is due to the presence of a STAT3 binding element in the hepcidin promoter [118].

It has been described that IL-6 and bone morphogenetic proteins (BMPs) control hepcidin secretion in different cancers such as breast and prostate cancers [33,119] and IL-6 levels were increased in lung cancer patients with poor prognosis [120]. In breast cancer, a study revealed that the architecture of the tumor and its microenvironment affect hepcidin regulatory pathways [121].

Hepcidin can bind to the extracellular loop of ferroportin, leading to the internalization of ferroportin in clathrin-coated wells and subsequent destruction in the lysosome [122]. In pancreatic cancer, increased hepcidin levels were correlated with tumor stage, with vascular invasion, and with poorer overall survival [67].

In a prostate cancer cell model, hepcidin contributed to cancer proliferation since it reduced the expression of ferroportin, thus resulting in increased Fe levels [35]. Both the hepcidin upregulation and ferroportin downregulation represent a reliable prognostic-independent biomarker in breast cancers [40]. The hepcidin–ferroportin axis plays a role in the development of cancers, particularly in the growth of tumors and metastases [123]. Decreased levels of ferroportin were also reported in prostate cancer, ovarian cancer, and myeloma [87,104,124]. The low level of ferroportin was a prognostic biomarker associated with a poor clinical outcome for myeloma patients [104]. Similarly, for prostate tissue, patients with benign prostatic hyperplasia had increased levels of ferroportin expression. On the contrary, decreased cytoplasmic ferroportin expression was correlated with an increase in the degree of malignancy as well as a decrease in the differentiation of prostate cancer cells. Hence, this suggests that the variation of protein expression levels is associated with the process of prostate cancer cell development [124]. Similarly, in adrenocortical carcinoma, a decrease in the expression of both ferroportin and ceruloplasmin was correlated with poor prognosis [66].

Lastly, ferroportin is an essential protein in cancer biology owing to numerous studies that have shown that genetic upregulation of ferroportin expression is sufficient to reduce the rate of proliferation in various cancers [125]. In conclusion, a decrease in ferroportin expression levels results in an increase in intracellular free Fe, thus increasing tumor cell aggressiveness [40,67,124].

Increased levels of ferritin, the main iron storage protein, are correlated with poor prognosis in high-grade serous ovarian cancers [87]. Increased ferritin is also found in testicular seminoma, glioblastoma, Hodgkin’s lymphoma, lung, colorectal, pancreatic, and breast cancers, which are also affected by this increase [21,42,53,68,75,76,83,96,113]. This ferritin increase is a reliable prognostic biomarker for ovary, lung, and breast cancers [43,52,87].

Several studies attempted to determine associations between variations in serum ferritin concentrations and cancer; however, discrepant results were obtained. On the one hand, increased serum ferritin concentrations were associated with shorter survival time and poor prognosis [44,54,55,62,63,77,84,93,100,101,110]. On the other hand, other studies failed to demonstrate any association between serum ferritin levels and cancer prognosis [126,127]. Such opposite results could be explained by the difficulty to standardize pre-analytical conditions, control specimens, or measurement procedures before serum ferritin quantitation. The L-chain of ferritin is predominant in serum (L-ferritin); however, an increase in the expression of H-ferritin mRNA has been observed in cancer cells [128]. Hence, H-ferritin could be a potential diagnostic biomarker for cancer detection [21].

As discussed previously, Fe is complexed with Tf in the blood, and it enters cells by binding to Tfr1. The expression levels of Tfr1 are increased in several cancer types, including glioma, lung, colorectal, pancreatic, breast, bladder, and ovarian cancers, but also hematological malignancies such as non-Hodgkin’s lymphoma and chronic lymphoid leukemia [45,53,65,69,80,85,87,97,109,129]. Moreover, Tfr1 expression may be correlated with tumor stage or cancer progression [129].

In lung cancer, the activation of EGFR induced the cellular redistribution of Tfr1 [130]. In colorectal cancer, the JAK/STAT pathway was involved in the downregulation of Tfr1, which promoted cancer progression. In this study, patients with decreased Tfr1 expression had decreased survival rate in contrast to patients with positive Tfr1 expression [80]. The increase in Tfr1 may be related to various oncogenes, such as c-myc, FBXL5 or the upregulation of IRP2 or HIF1 [18,125].

Tfr2 is upregulated in cancer cells and glioblastoma [131]. Such an increased expression level represents a favorable prognostic in glioblastoma [99]. Moreover, it is important to note that increased transferrin saturation is not only a risk factor for cancer initiation [125] but also a bad prognostic marker associated with increased mortality [126,132,133].

Aside from the canonical transferrin source of Fe, cells can also obtain Fe via a secondary and less studied lipocalin-based pathway. Lipocalin-2 (LCN2) forms a complex with Fe that is internalized after specific interaction with cell surface receptors. The LCN2 protein also participates in the immune system because it catches Fe complexed with bacterial siderophores. Therefore, it prevents bacteria from acquiring the Fe necessary for their growth [134]. Lipocalin-2 can be either increased or decreased in different cancers [135], affecting the final prognosis [64,135].

Lipocalin is upregulated in different cancers, such as lung cancer. In the lung, increased levels of lipocalin are associated with radio-responsiveness and this protein could serve as an early-stage biomarker [56,57]. Direct measurements of serum Fe concentration were evaluated for possible association with cancer; however, results were not always consistent between studies [136,137,138,139].

The risk of developing cancer was greater when serum Fe concentrations were outside reference intervals, i.e., below 60 or over 120 µg/dL [140]. In a contradictory study, the increase in serum Fe concentration reduced the risk for cancer [141]. Importantly, since inflammation disturbs the normal Fe homeostatic mechanisms and induces the redistribution of Fe, studies aiming at determining the link between serum Fe levels and cancer disease should take into account the presence of potential and concomitant inflammatory reactions in cancer patients [142].

Several studies attempted to find correlations between Fe homeostasis gene signatures and the prognosis of cancers. In breast cancer, four Fe homeostasis genes (namely CYBRD1/DCYTB, LTF, STEAP1, and STEAP2) had significantly reduced expression levels in metastasis compared to primary tumors [46].

Among genes from Fe-related metabolism, a specific gene signature was able to discriminate between liver cancer and adjacent non-tumor tissues. Effectively, in HCC tumors, the increased expression levels of Fe-related FLVCR1 and TFRC genes were associated with various factors leading to poor prognosis such as vascular invasion and the histological grade of the tumor for FLVCR1. In fact, TFRC encodes transferrin receptor 1, and feline leukemia virus subgroup C receptor 1 (encoded by FLVCR1) is a protein that helps in preventing oxidative damage due to excess iron [60].

In silico mining of proteomic and epigenetic data from The Cancer Genome Atlas (TCGA) database allowed the identification of iron-related gene alterations in 14 cancers. The expression of Cp was increased in six types of cancer, while Cp was decreased in three types of cancer. These dysregulations also affected other actors in iron homeostasis such as Tfr2, LCN2, TFRC, and CYBRD1/DCYTB, sometimes being associated with patient survival [39].

The expression levels of the STEAP family of metalloreductases are also altered in some cancers [143].

STEAP3 protein is a p53 inducible protein [144] which induces apoptotic cell death via a caspase-3 dependent pathway [145]. In prostate cancer, STEAP1 and STEAP2 proteins are upregulated. The increase in STEAP2 and its localization is associated with the aggressiveness of the tumor. This suggests that STEAP2 and possibly STEAP1 could serve as prognostic biomarkers in oncology [36,37,38]. Increased STEAP3 expression levels were also observed in glioblastoma and this pattern was associated with reduced overall survival [98]. In addition, cancerous colorectal tissue had higher STEAP3 mRNA expression and Fe storage compared to healthy colon tissue. In this study, it was suggested that STEAP3 had a role not only in Fe storage in cancer cells but also in tumor proliferation under hypoferric conditions [146].

DMT1 may also contribute to colorectal cancer progression and the increased expression of DCYTB and DMT1 was correlated with advanced tumor stages [147,148]. Similarly, in breast cancer, increased levels of DCYTB were associated with prolonged survival and response to treatment [149].

Finally, miRNAs are frequently dysregulated in cancers [150]. In different cancers, the altered expression levels of miRNAs impacted the iron intake and/or its metabolism [23]. In hepatocellular carcinoma (HCC), decreased miR-148a levels were associated with Tfr1 mRNA levels [151] and the downregulation of miR-152 may induce increases in Tfr1 levels [152]. Similarly, in lung adenocarcinoma and lung squamous cell carcinoma, the increased expression of miR-20 induced a decrease in ferroportin mRNA expression levels, which resulted in Fe retention and increased proliferation [51]. In breast cancer patients, miR-320 expression was decreased in plasma and tumor tissue [153]. In addition, an increased level of miR-320 may repress the expression of Tfr1 and lead to inhibition of cell proliferation [154]. In multiple myeloma, miR-17-5p was identified as a regulator of ferroportin in vitro and in vivo, leading to increased cell proliferation and inhibition of apoptosis [155].

## 4. Iron as a Target or a Bullet for Cancer Treatment

There is a strong rationale for targeting Fe and its metabolism to fight against cancer proliferation. Several therapeutic strategies are currently being investigated, such as the use of chelators to limit the availability of Fe to cancer cells, or strategies exploiting the redox properties of excess Fe, or the intense oxidative stress of ferroptosis. In this section, we will review the four main anticancer therapeutic approaches targeting Fe metabolism.

### 4.1. Fe Chelators

The first Fe chelators were developed in the 1990s. This 30-year-old strategy is based on the strong dependence of cancer cells on Fe, which is, indeed, an essential element for cell proliferation and DNA replication in cancer cells [156]. Iron chelators reduce Fe intake, which alters the metabolism of cancer cells. Iron chelators also inhibit ribonucleotide reductase activity and act on multiple signaling pathways related to tumor progression and metastatic development [157,158].

The application of chelating agents seems to be effective only on specific cancers. Deferoxamine (DFO) chelator is effective in hepatocellular carcinoma, whereas it is ineffective in recurrent neuroblastoma [159,160]. Iron chelators that act on the redox cycle are considered promising antitumor agents [161]. Currently, DFO and triapine are chelators that are already tested for human cancers in clinical trials (NCT02466971) [162].

Numerous synthetic Fe chelators were developed to increase their efficacy while reducing side effects and unwanted toxicity, and also for developing the per os route of administration [163,164]. Chelators are frequently used in combination with other therapies. As an example, DFO was associated with many different chemotherapeutic drugs such as cyclophosphamide, thiotepa, etoposide, carboplatin, and cisplatin [165,166,167]. However, some cancers are resistant to Fe chelators, probably because of their low membrane permeability properties [163]. Despite these limitations, the interest in DFO and Fe chelators in general has not disappeared in view of the ongoing clinical trials and research efforts focusing on the optimization of their vectorization [168].

### 4.2. Fe-Containing Molecules

Another therapeutic strategy targeting Fe metabolism is based on the use of metal-containing drugs, also named bio-organometallic molecules. Organometallic Fe compounds such as ferrocene are a very large group of substances, interesting for the design of biologically active molecules. Ferrocene derivatives have anti-proliferative activity against several cancer cell lines [169]. In addition, they are stable in aqueous solution and display advantageous redox properties with low toxicity [170,171].

### 4.3. Fe Metabolism Disruptors

The third strategy is to directly target the proteins involved in dysregulated Fe metabolism or to exploit their physicochemical properties to develop new therapeutics.

Gallium is a group IIIa metal with shared chemical characteristics with iron. It is therefore possible to mimic the properties of Fe by using gallium (Ga) to disrupt Fe metabolism. Gallium is bound into Fe active pockets of ribonucleotide reductase, which blocks enzymatic reactions and leads to increased reactive mitochondrial oxygen species in cells. Gallium-based compounds disrupt iron-dependent tumor metabolism and have antineoplastic activity [172].

In the aim of targeting Fe proteins, strategies against Tfr1 receptor appear promising since the ligand–receptor interface can be targeted by antibodies mimicking transferrin. This interaction will reduce the amount of Fe entering the cell via Tfr1. The Tfr1 protein can also be used as a vector to deliver cytotoxic antitumor agents inside cells [129]. Transferrin is an excellent conjugation partner for chemotherapy due to its high affinity for Tfr1 membrane receptor, which is frequently overexpressed in cancer cells. Some drugs target the hepcidin–ferroportin axis that allows Fe efflux from cells, thus disrupting the Fe metabolism of cancer cells. Other parallel techniques are currently being studied, such as disruption of the BMP/HJV/SMAD pathway, the use of microRNA, and disruption of the hepcidin–ferroportin interaction [173].

Ferritin is a major iron storage protein that can easily be synthesized and mineralized for the production of nanoparticles (NPs) such as magnetoferritin. This nano-object has theranostic properties, i.e., it can be used as an imaging probe to visualize the tumor and it is also an active compound that targets it. For therapeutic drug delivery purposes, ferritin NPs have also been designed to encapsulate chemical drugs [128].

Interestingly, the use of miRNAs alone or in combination with drugs as tools and/or targets is an emerging therapeutic strategy for cancer [174,175]. The transfection of miR-200b decreased the levels of ferritin in vitro and increased the sensitivity of cancer cells to the chemotherapeutic agent doxorubicin [176].

### 4.4. Ferroptosis Inducers

Especially developed for fighting against the emergence of resistance mechanisms [177], the fourth strategy is aimed at inducing ferroptosis, which is a type of programmed cell death dependent on iron. The intracellular Fe concentration and the lipid peroxidation are two critical biochemical parameters for autophagy-dependent ferroptosis. Ferroptosis differs from other controlled cell death processes such as apoptosis, especially by cell morphology and the underlying genetic and biochemical mechanisms. RAS oncogene is known to be an inducer of ferroptosis, and TP53 tumor suppressor gene may also participate in this cell death mechanism [178,179]. Epigenetic regulatory elements can modulate the induction mechanisms of ferroptosis and oncogenesis [180,181]. 

Various inducers of ferroptosis have been discovered. These compounds alter membrane permeability and cause major perturbations of cellular redox metabolism, leading to altered cell viability. Recently, the intracellular impact of salinomycin derivatives has been characterized [182]. In fact, these molecules sequester Fe in lysosomes, leading to Fe cytosolic depletion and lysosomal membrane permeabilization. This iron homeostasis disruption thus triggers ferroptosis cell death in a very efficient way, since it is able to kill cancer stem cells, which are generally considered refractory to conventional anticancer treatments. 

Another example is the use of Fe oxide NPs to induce ferroptosis in cancer cells by increasing iron levels and ROS production, and the American Food and Drug Administration has already given approval for the clinical use of ferroptosis-inducing compounds, which are deemed promising molecules in the treatment of resistant cancers [180].

## 5. Conclusions about Iron and Cancer

Iron metabolism is disrupted in a number of diseases, including cancer. Cancer cells have increased Fe requirements and alterations of Fe homeostasis allow cancer cells to maintain a high proliferation rate. However, this dependence on Fe also represents an Achilles’ heel for cancer cells, and targeting Fe metabolism is a relevant and promising anticancer strategy. According to cancer types, the increased or decreased levels of different actors in Fe metabolism may serve as promising predictive or prognostic biomarkers. Improved knowledge of Fe dysregulation pathways prompted the development of therapeutic strategies targeting Fe. Some of the most advanced agents are even prescribed to cancer patients within clinical trials. Ideally, the combination of Fe-targeting drugs with chemotherapeutic molecules should be preferred, since it may result in cumulative or synergistic anti-proliferative effects.

## Figures and Tables

**Table 1:** Fe regulators and their roles in cancer (+: upregulated; −: downregulated).

Cancer	Altered Player	Regulation	Sample	Prognostic	Ref.
Prostate Adenocarcinoma	Hepcidin	+	Tissue	poor	[33]
Prostate Adenocarcinoma	Hepcidin	+	Systemic (liver) hepcidin expression		[34]
Prostate Adenocarcinoma	Ferroportin	−	Tissue	poor	[35]
Prostate Adenocarcinoma	STEAP1	+	Tissue	poor	[36]
Prostate Adenocarcinoma	STEAP2	+	Tissue	poor	[37,38]
Prostate Adenocarcinoma	HFE	−	Tissue		[39]
Breast Invasive Carcinoma	Hepcidin	+	Tissue	poor	[40]
Breast Invasive Carcinoma	Hepcidin	+	Systemic (liver) hepcidin expression		[41]
Breast Invasive Carcinoma	Ferroportin	−	Tissue	poor	[40]
Breast Invasive Carcinoma	Ferritin	+	Tissue	poor	[42,43]
Breast Invasive Carcinoma	Ferritin	+	Serum	poor	[44]
Breast Invasive Carcinoma	Tfr1	+	Tissue	poor	[45]
Breast Invasive Carcinoma	STEAP1	−	Tissue		[46]
Breast Invasive Carcinoma	STEAP2	−	Tissue		[46]
Breast Invasive Carcinoma	LTF	−	Tissue		[46]
Breast Invasive Carcinoma	CYBRD1	−	Tissue		[46]
Breast Invasive Carcinoma	Lipocalin 2	+	Tissue	poor	[47,48,49]
Breast Invasive Carcinoma	ERFE	+	Tissue		[39]
Breast Invasive Carcinoma	FLVCR1	+	Tissue		[39]
Breast Invasive Carcinoma	Tf	−	Tissue		[39]
Breast Invasive Carcinoma	Tfr2	+	Tissue		[39]
Breast Invasive Carcinoma	Tfr1	+	Tissue		[39]
Non-Small-Cell Lung Carcinoma	Hepcidin	+	Tissue	poor	[50]
Non-Small-Cell Lung Carcinoma	Hepcidin	+	Systemic (liver) hepcidin expression	poor	[50]
Lung Adenocarcinoma/Lung Squamous Cell Carcinoma	Ferroportin	−	Tissue	poor	[51]
Lung/Non-Small-Cell Lung Cancer	Ferritin	+	Tissue	poor	[52,53]
Lung/Non-Small Cell Lung Cancer	Ferritin	+	Serum	poor	[54,55]
Non-Small Cell Lung Cancer	Tfr1	+	Tissue		[53]
Lung Squamous Cell Carcinoma	Tfr1	+	Tissue		[39]
Oral Squamous Cell Carcinoma/Non-Small Cell Lung Cancer	Lipocalin 2	+	Tissue	poor	[56,57]
Lung Adenocarcinoma	ERFE	+	Tissue		[39]
Lung Squamous Cell Carcinoma		+	Tissue		[39]
Lung Adenocarcinoma	FLVCR1	+	Tissue		[39]
Lung Adenocarcinoma	Cp	+	Tissue		[39]
Lung Adenocarcinoma	Tfr2	+	Tissue		[39]
Lung Squamous Cell Carcinoma	Tfr2	+	Tissue		[39]
Lung Squamous Cell Carcinoma	STEAP4	−	Tissue		[39]
Lung Adenocarcinoma	STEAP3	+	Tissue		[39]
Lung Adenocarcinoma	STEAP1	+	Tissue		[39]
Lung Squamous Cell Carcinoma		+	Tissue		[39]
Lung Adenocarcinoma	CYBRD1	−	Tissue		[39]
Lung Squamous Cell Carcinoma		−	Tissue		[39]
Liver Hepatocellular Carcinoma	Hepcidin	−	Tissue		[58]
Liver Hepatocellular Carcinoma	Hepcidin	−	Tissue		[59]
Liver Hepatocellular Carcinoma	Hepcidin	−	Systemic (liver) hepcidin expression		[59]
Liver Hepatocellular Carcinoma	Tfr1	+	Tissue		[60]
Liver Hepatocellular Carcinoma	FLVCR1	+	Tissue		[60]
Liver Hepatocellular Carcinoma	HMOX1	−	Tissue		[60]
Liver Hepatocellular Carcinoma	SLC25A37	−	Tissue		[60]
Liver Hepatocellular Carcinoma	SLC25A38	−	Tissue	poor	[60]
Liver Hepatocellular Carcinoma	FTH1	+	Tissue		[60]
Liver Hepatocellular Carcinoma	FTL	+/−	Tissue	poor/good	[60]
Liver Hepatocellular Carcinoma	ERFE	+	Tissue		[39]
Liver Hepatocellular Carcinoma	FLVCR1	+	Tissue		[39]
Liver Hepatocellular Carcinoma	STEAP3	−	Tissue		[39]
Liver Hepatocellular Carcinoma	STEAP4	−	Tissue		[39]
Hepatocellular Cancer	Lipocalin 2	+	Tissue		[61]
Renal Carcinoma	Hepcidin	+	Tissue	poor	[62]
Renal Carcinoma	Ferritin	+	Serum	poor	[63]
Kidney Renal Clear Cell Carcinoma	Lipocalin 2	+	Tissue	poor	[64]
Kidney Chromophobe	Cp	−	Tissue		[39]
Kidney Renal Clear Cell Carcinoma	Cp	+	Tissue		[39]
Kidney Renal Papillary Cell Carcinoma	Tfr2	+	Tissue		[39]
Kidney Renal Clear Cell Carcinoma	Tfr2	+	Tissue		[39]
Kidney Chromophobe	FTH1	+	Tissue		[39]
Kidney Renal Papillary Cell Carcinoma	HAMP	+	Tissue		[39]
Kidney Renal Clear Cell Carcinoma	HAMP	+	Tissue		[39]
Kidney Renal Clear Cell Carcinoma	STEAP3	+	Tissue		[39]
Bladder Cancer	Tfr1	+	Tissue	poor	[65]
Adrenocortical Carcinoma	Ferroportin	−	Tissue	poor	[66]
Pancreatic Cancer	Hepcidin	Variable	Tissue		[67]
Pancreatic Cancer	Ferroportin	−	Tissue	poor	[67]
Pancreatic Cancer	Ferritin	+	Tissue		[68]
Pancreatic Adenocarcinoma	Tfr1	+	Tissue	poor	[69]
Pancreatic Adenocarcinoma	Lipocalin 2	+	Tissue	Discrepancies	[70,71,72,73,74]
Colorectal Cancer	Ferritin	+	Tissue		[75]
Colon Adenocarcinoma	Ferritin	+	Tissue		[76]
Colorectal Cancer	Ferritin	+	Serum	poor	[77]
Colorectal Cancer	Lipocalin 2	+	Tissue	poor	[78,79]
Colorectal Cancer	Tfr1	+	Tissue	good	[80]
Colon Adenocarcinoma	ERFE	+	Tissue		[39]
Colon Adenocarcinoma	Cp	−	Tissue		[39]
Colon Adenocarcinoma	Tfr2	+	Tissue		[39]
Colon Adenocarcinoma	CYBRD1	−	Tissue		[39]
Rectum Adenocarcinoma	Lipocalin 2	+	Tissue	poor	[81]
Hodgkin’s Lymphoma	Hepcidin	+	Tissue		[82]
Hodgkin’s Lymphoma	Ferritin	+	Tissue		[83]
Hodgkin’s Lymphoma	Ferritin	+	Serum	poor	[84]
Non Hodgkin’s Lymphoma	Tfr1	+	Tissue	poor	[85]
Non Hodgkin’s Lymphoma	Hepcidin	+	Tissue		[86]
Non Hodgkin’s Lymphoma	Hepcidin	+	Systemic (liver) hepcidin expression		[86]
Ovarian Cancer	Ferroportin	−	Tissue	poor	[87]
Ovarian Cancer	Ferritin	+	Tissue	poor	[87]
Ovarian Cancer	Tfr1	+	Tissue	poor	[87]
Ovarian	Lipocalin 2	+	Tissue	poor	[88,89,90]
Uterine Corpus Endometrial Carcinoma	ERFE	+	Tissue		[39]
Uterine Corpus Endometrial Carcinoma	FLVCR1	+	Tissue		[39]
Uterine Corpus Endometrial Carcinoma	Tfr2	+	Tissue		[39]
Uterine Corpus Endometrial Carcinoma	CYBRD1	−	Tissue		[39]
Uterine Corpus Endometrial Carcinoma	STEAP4	−	Tissue		[39]
Endometrium	Lipocalin 2	+	Tissue	good	[91,92]
Cervical Cancer	Ferritin	+	Serum	poor	[93]
Brain	Hepcidin	−	Tissue		[94]
Brain	Lipocalin 2	+	Tissue	poor	[95]
Glioblastoma	Ferritin	+	Tissue		[96]
Glioma	Tfr1	+	Tissue	poor	[97]
Glioblastoma	STEAP3	+	Tissue	poor	[98]
Gliobastoma	Tfr2	+	Tissue	good	[99]
Neuroblastoma	Ferritin	+	Serum	poor	[100]
Lymphoma T-cell	Ferritin	+	Serum	poor	[101]
Head and Neck squamous Cell Carcinoma	Tf	−	Tissue		[39]
Head And Neck Squamous Cell Carcinoma	Tfr1	+	Tissue		[39]
Multiple Myeloma	Hepcidin	−	Tissue		[102]
Multiple Myeloma	Hepcidin	+	Systemic (liver) hepcidin expression		[103]
Myeloma	Ferroportin	−	Tissue	poor	[104]
Thyroid Carcinoma	Lipocalin 2	+	Tissue	good	[105]
Thyroid Carcinoma	Hepcidin	+	Tissue		[106]
Leukaemia	Hepcidin	+	Systemic (liver) hepcidin expression		[107]
Chronic Myelogenous Leukemia	Lipocalin 2	+	Tissue	poor	[108]
Chronic Lymphocytic Leukemia	Tfr1	+	Tissue	poor	[109]
Oral Squamous Carcinoma	Ferritin	+	Serum	poor	[110]
Oesophageal Cell Carcinoma	Lipocalin 2	+	Tissue	poor	[111]
Stomach Adenocarcinoma	CYBRD1	−	Tissue		[39]
Stomach Adenocarcinoma	Lipocalin 2	+	Tissue	poor	[108,112]
Testicular Seminoma	Ferritin	+	Tissue		[113]

## References

[1] (2016). Intracellular Iron Utilisation. Iron Metabolism.

[2] Srai S.K., Sharp P., Anderson G.J., McLaren G.D. (2012). Proteins of Iron Homeostasis. Iron Physiology and Pathophysiology in Humans.

[3] Zhang D.-L., Ghosh M.C., Rouault T.A. (2014). The physiological functions of iron regulatory proteins in iron homeostasis-an update. Front. Pharmacol..

[4] Ganz T., Vaulont S., Anderson G.J., McLaren G.D. (2012). Molecular Regulation of Systemic Iron Metabolism. Iron Physiology and Pathophysiology in Humans.

[5] Ruddell R.G., Ramm G.A., Anderson G.J., McLaren G.D. (2012). Hepatic Pathobiology of Iron Overload. Iron Physiology and Pathophysiology in Humans.

[6] Winterbourn C.C. (1995). Toxicity of iron and hydrogen peroxide: The Fenton reaction. Toxicol. Lett..

[7] Muir A., Hopfer U. (1985). Regional specificity of iron uptake by small intestinal brush-border membranes from normal and iron-deficient mice. Am. J. Physiol. Gastrointest. Liver Physiol..

[8] McKie A.T., Simpson R.J., Anderson G.J., McLaren G.D. (2012). Intestinal Iron Absorption. Iron Physiology and Pathophysiology in Humans.

[9] Pantopoulos K. (2004). Iron Metabolism and the IRE/IRP Regulatory System: An Update. Ann. N. Y. Acad. Sci..

[10] Iolascon A., De Falco L. (2009). Mutations in the Gene Encoding DMT1: Clinical Presentation and Treatment. Semin. Hematol..

[11] Hooda J., Shah A., Zhang L. (2014). Heme, an Essential Nutrient from Dietary Proteins, Critically Impacts Diverse Physiological and Pathological Processes. Nutrients.

[12] McKie A.T., Marciani P., Rolfs A., Brennan K., Wehr K., Barrow D., Miret S., Bomford A., Peters T.J., Farzaneh F. (2000). A Novel Duodenal Iron-Regulated Transporter, IREG1, Implicated in the Basolateral Transfer of Iron to the Circulation. Mol. Cell.

[13] Donovan A., Lima C.A., Pinkus J.L., Pinkus G.S., Zon L.I., Robine S., Andrews N.C. (2005). The iron exporter ferroportin/Slc40a1 is essential for iron homeostasis. Cell Metab..

[14] Liu X., Hill P., Haile D.J. (2002). Role of the Ferroportin Iron-Responsive Element in Iron and Nitric Oxide Dependent Gene Regulation. Blood Cells Mol. Dis..

[15] Reichert C.O., da Cunha J., Levy D., Maselli L.M.F., Bydlowski S.P., Spada C. (2017). Hepcidin: Homeostasis and Diseases Related to Iron Metabolism. Acta Haematol..

[16] Linder M.C. (2016). Ceruloplasmin and other copper binding components of blood plasma and their functions: An update. Metallomics.

[17] Kawabata H. (2019). Transferrin and transferrin receptors update. Free Radic. Biol. Med..

[18] Torti S.V., Torti F.M. (2013). Iron and cancer: More ore to be mined. Nat. Rev. Cancer.

[19] Ohgami R.S., Campagna D.R., McDonald A., Fleming M.D. (2006). The Steap proteins are metalloreductases. Blood.

[20] Ohgami R.S., Campagna D.R., Greer E.L., Antiochos B., McDonald A., Chen J., Sharp J.J., Fujiwara Y., Barker J.E., Fleming M.D. (2005). Identification of a ferrireductase required for efficient transferrin-dependent iron uptake in erythroid cells. Nat. Genet..

[21] Torti F.M., Torti S.V. (2002). Regulation of ferritin genes and protein. Blood.

[22] Manz D.H., Blanchette N.L., Paul B.T., Torti F.M., Torti S.V. (2016). Iron and cancer: Recent insights. Ann. N. Y. Acad. Sci..

[23] Wang Y., Yu L., Ding J., Chen Y. (2018). Iron Metabolism in Cancer. Int. J. Mol. Sci..

[24] Hirschhorn T., Stockwell B.R. (2019). The development of the concept of ferroptosis. Free Radic. Biol. Med..

[25] Phaniendra A., Jestadi D.B., Periyasamy L. (2015). Free Radicals: Properties, Sources, Targets, and Their Implication in Various Diseases. Indian J. Clin. Biochem..

[26] Guo H.-F., Tsai C.-L., Terajima M., Tan X., Banerjee P., Miller M.D., Liu X., Yu J., Byemerwa J., Alvarado S. (2018). Pro-metastatic collagen lysyl hydroxylase dimer assemblies stabilized by Fe 2+ -binding. Nat. Commun..

[27] Gobin E., Bagwell K., Wagner J., Mysona D., Sandirasegarane S., Smith N., Bai S., Sharma A., Schleifer R., She J.-X. (2019). A pan-cancer perspective of matrix metalloproteases (MMP) gene expression profile and their diagnostic/prognostic potential. BMC Cancer.

[28] Puig S., Ramos-Alonso L., Romero A.M., Martínez-Pastor M.T. (2017). The elemental role of iron in DNA synthesis and repair. Metallomics.

[29] Tong W.-H., Maio N., Zhang D.-L., Palmieri E.M., Ollivierre H., Ghosh M.C., McVicar D.W., Rouault T.A. (2018). TLR-activated repression of Fe-S cluster biogenesis drives a metabolic shift and alters histone and tubulin acetylation. Blood Adv..

[30] Cao L.-L., Liu H., Yue Z., Liu L., Pei L., Gu J., Wang H., Jia M. (2018). Iron chelation inhibits cancer cell growth and modulates global histone methylation status in colorectal cancer. Biometals.

[31] Huang Y., Rao A. (2014). Connections between TET proteins and aberrant DNA modification in cancer. Trends Genet..

[32] Torti S.V., Manz D.H., Paul B.T., Blanchette-Farra N., Torti F.M. (2018). Iron and Cancer. Annu. Rev. Nutr..

[33] Tesfay L., Clausen K.A., Kim J.W., Hegde P., Wang X., Miller L.D., Deng Z., Blanchette N., Arvedson T., Miranti C.K. (2015). Hepcidin Regulation in Prostate and Its Disruption in Prostate Cancer. Cancer Res..

[34] Tanno T., Rabel A., Alleyne M., Lee Y.T., Dahut W.L., Gulley J.L., Miller J.L. (2011). Hepcidin, anaemia, and prostate cancer: Letters. BJU Int..

[35] Zhao B., Li R., Cheng G., Li Z., Zhang Z., Li J., Zhang G., Bi C., Hu C., Yang L. (2018). Role of hepcidin and iron metabolism in the onset of prostate cancer. Oncol. Lett..

[36] Kim K., Mitra S., Wu G., Berka V., Song J., Yu Y., Poget S., Wang D.-N., Tsai A.-L., Zhou M. (2016). Six-Transmembrane Epithelial Antigen of Prostate 1 (STEAP1) Has a Single *b* Heme and Is Capable of Reducing Metal Ion Complexes and Oxygen. Biochemistry.

[37] Whiteland H., Spencer-Harty S., Morgan C., Kynaston H., Thomas D.H., Bose P., Fenn N., Lewis P., Jenkins S., Doak S.H. (2014). A role for STEAP2 in prostate cancer progression. Clin. Exp. Metastasis.

[38] Burnell S.E.A., Spencer-Harty S., Howarth S., Bodger O., Kynaston H., Morgan C., Doak S.H. (2018). STEAP2 Knockdown Reduces the Invasive Potential of Prostate Cancer Cells. Sci. Rep..

[39] Zhang S., Chang W., Wu H., Wang Y., Gong Y., Zhao Y., Liu S., Wang H., Svatek R.S., Rodriguez R. (2020). Pan-cancer analysis of iron metabolic landscape across the Cancer Genome Atlas. J. Cell Physiol..

[40] Pinnix Z.K., Miller L.D., Wang W., D’Agostino R., Kute T., Willingham M.C., Hatcher H., Tesfay L., Sui G., Di X. (2010). Ferroportin and Iron Regulation in Breast Cancer Progression and Prognosis. Sci. Transl. Med..

[41] Ciniselli C.M., De Bortoli M., Taverna E., Varinelli L., Pizzamiglio S., Veneroni S., Bonini C., Orlandi R., Verderio P., Bongarzone I. (2015). Plasma hepcidin in early-stage breast cancer patients: No relationship with interleukin-6, erythropoietin and erythroferrone. Expert Rev. Proteom..

[42] Weinstein R.E., Bond B.H., Silberberg B.K. (1982). Tissue ferritin concentration in carcinoma of the breast. Cancer.

[43] Alkhateeb A.A., Han B., Connor J.R. (2013). Ferritin stimulates breast cancer cells through an iron-independent mechanism and is localized within tumor-associated macrophages. Breast Cancer Res. Treat..

[44] Marcus D.M., Zinberg N. (1975). Measurement of serum ferritin by radioimmunoassay: Results in normal individuals and patients with breast cancer. J. Natl. Cancer Inst..

[45] Habashy H.O., Powe D.G., Staka C.M., Rakha E.A., Ball G., Green A.R., Aleskandarany M., Paish E.C., Douglas Macmillan R., Nicholson R.I. (2010). Transferrin receptor (CD71) is a marker of poor prognosis in breast cancer and can predict response to tamoxifen. Breast Cancer Res. Treat..

[46] Miller L.D., Coffman L.G., Chou J.W., Black M.A., Bergh J., D’Agostino R., Torti S.V., Torti F.M. (2011). An Iron Regulatory Gene Signature Predicts Outcome in Breast Cancer. Cancer Res..

[47] Bauer M., Eickhoff J.C., Gould M.N., Mundhenke C., Maass N., Friedl A. (2008). Neutrophil gelatinase-associated lipocalin (NGAL) is a predictor of poor prognosis in human primary breast cancer. Breast Cancer Res. Treat..

[48] Nacht M., Ferguson A.T., Zhang W., Petroziello J.M., Cook B.P., Gao Y.H., Maguire S., Riley D., Coppola G., Landes G.M. (1999). Combining serial analysis of gene expression and array technologies to identify genes differentially expressed in breast cancer. Cancer Res..

[49] Stoesz S.P., Friedl A., Haag J.D., Lindstrom M.J., Clark G.M., Gould M.N. (1998). Heterogeneous expression of the lipocalin NGAL in primary breast cancers. Int. J. Cancer.

[50] Chen Q., Wang L., Ma Y., Wu X., Jin L., Yu F. (2014). Increased hepcidin expression in non-small cell lung cancer tissue and serum is associated with clinical stage: Increased hepcidin expression in NSCLC. Thorac. Cancer.

[51] Babu K.R., Muckenthaler M.U. (2016). miR-20a regulates expression of the iron exporter ferroportin in lung cancer. J. Mol. Med..

[52] Sukiennicki G.M., Marciniak W., Muszyńska M., Baszuk P., Gupta S., Białkowska K., Jaworska-Bieniek K., Durda K., Lener M., Pietrzak S. (2019). Iron levels, genes involved in iron metabolism and antioxidative processes and lung cancer incidence. PLoS ONE.

[53] Kukulj S., Jaganjac M., Boranic M., Krizanac S., Santic Z., Poljak-Blazi M. (2010). Altered iron metabolism, inflammation, transferrin receptors, and ferritin expression in non-small-cell lung cancer. Med. Oncol..

[54] Ferrigno D., Buccheri G. (1992). Serum ferritin levels in lung cancer patients. Eur. J. Cancer.

[55] Lee S., Eo W., Jeon H., Park S., Chae J. (2017). Prognostic Significance of Host-related Biomarkers for Survival in Patients with Advanced Non-Small Cell Lung Cancer. J. Cancer.

[56] Sun B., Guo W., Hu S., Yao F., Yu K., Xing J., Wang R., Song H., Liao Y., Wang T. (2017). Gprc5a-knockout mouse lung epithelial cells predicts ceruloplasmin, lipocalin 2 and periostin as potential biomarkers at early stages of lung tumorigenesis. Oncotarget.

[57] Shiiba M., Saito K., Fushimi K., Ishigami T., Shinozuka K., Nakashima D., Kouzu Y., Koike H., Kasamatsu A., Sakamoto Y. (2013). Lipocalin-2 is associated with radioresistance in oral cancer and lung cancer cells. Int. J. Oncol..

[58] Kijima H., Sawada T., Tomosugi N., Kubota K. (2008). Expression of hepcidin mRNA is uniformly suppressed in hepatocellular carcinoma. BMC Cancer.

[59] Kessler S.M., Laggai S., Kiemer A.K., Barghash A., Helms V. (2015). Hepatic hepcidin expression is decreased in cirrhosis and HCC. J. Hepatol..

[60] Shen Y., Li X., Zhao B., Xue Y., Wang S., Chen X., Yang J., Lv H., Shang P. (2018). Iron metabolism gene expression and prognostic features of hepatocellular carcinoma: SHEN. J. Cell Biochem..

[61] Lee E.K., Kim H.J., Lee K.J., Lee H.J., Lee J.S., Kim D.G., Hong S.W., Yoon Y., Kim J.S. (2011). Inhibition of the proliferation and invasion of hepatocellular carcinoma cells by lipocalin 2 through blockade of JNK and PI3K/Akt signaling. Int. J. Oncol..

[62] Kamai T., Tomosugi N., Abe H., Arai K., Yoshida K.-I. (2009). Increased serum hepcidin-25 level and increased tumor expression of hepcidin mRNA are associated with metastasis of renal cell carcinoma. BMC Cancer.

[63] Kirkali Z., Güzelsoy M., Mungan M.U., Kirkali G., Yörükoglu K. (1999). Serum ferritin as a clinical marker for renal cell carcinoma: Influence of tumor size and volume. Urol. Int..

[64] Rehwald C., Schnetz M., Urbschat A., Mertens C., Meier J.K., Bauer R., Baer P., Winslow S., Roos F.C., Zwicker K. (2020). The iron load of lipocalin-2 (LCN-2) defines its pro-tumour function in clear-cell renal cell carcinoma. Br. J. Cancer.

[65] Seymour G.J., Walsh M.D., Lavin M.F., Strutton G., Gardiner R.A. (1987). Transferrin receptor expression by human bladder transitional cell carcinomas. Urol. Res..

[66] Zhu B., Zhi Q., Xie Q., Wu X., Gao Y., Chen X., Shi L. (2019). Reduced expression of ferroportin1 and ceruloplasmin predicts poor prognosis in adrenocortical carcinoma. J. Trace Elem. Med. Biol..

[67] Toshiyama R., Konno M., Eguchi H., Asai A., Noda T., Koseki J., Asukai K., Ohashi T., Matsushita K., Iwagami Y. (2018). Association of iron metabolic enzyme hepcidin expression levels with the prognosis of patients with pancreatic cancer. Oncol. Lett..

[68] Marcus D.M., Zinberg N. (1974). Isolation of ferritin from human mammary and pancreatic carcinomas by means of antibody immunoadsorbents. Arch. Biochem. Biophys..

[69] Jeong S.M., Hwang S., Seong R.H. (2016). Transferrin receptor regulates pancreatic cancer growth by modulating mitochondrial respiration and ROS generation. Biochem. Biophys. Res. Commun..

[70] Argani P., Rosty C., Reiter R.E., Wilentz R.E., Murugesan S.R., Leach S.D., Ryu B., Skinner H.G., Goggins M., Jaffee E.M. (2001). Discovery of new markers of cancer through serial analysis of gene expression: Prostate stem cell antigen is overexpressed in pancreatic adenocarcinoma. Cancer Res..

[71] Laurell H. (2006). Identification of biomarkers of human pancreatic adenocarcinomas by expression profiling and validation with gene expression analysis in endoscopic ultrasound-guided fine needle aspiration samples. World J. Gastroenterol. WJG.

[72] Moniaux N., Chakraborty S., Yalniz M., Gonzalez J., Shostrom V.K., Standop J., Lele S.M., Ouellette M., Pour P.M., Sasson A.R. (2008). Early diagnosis of pancreatic cancer: Neutrophil gelatinase-associated lipocalin as a marker of pancreatic intraepithelial neoplasia. Br. J. Cancer.

[73] Tong Z., Kunnumakkara A.B., Wang H., Matsuo Y., Diagaradjane P., Harikumar K.B., Ramachandran V., Sung B., Chakraborty A., Bresalier R.S. (2008). Neutrophil Gelatinase-Associated Lipocalin: A Novel Suppressor of Invasion and Angiogenesis in Pancreatic Cancer. Cancer Res..

[74] Furutani M., Arii S., Mizumoto M., Kato M., Imamura M. (1998). Identification of a neutrophil gelatinase-associated lipocalin mRNA in human pancreatic cancers using a modified signal sequence trap method. Cancer Lett..

[75] Sornjai W., Nguyen Van Long F., Pion N., Pasquer A., Saurin J.-C., Marcel V., Diaz J.J., Mertani H.C., Smith D.R. (2020). Iron and hepcidin mediate human colorectal cancer cell growth. Chem. Biol. Interact..

[76] Vaughn C.B., Weinstein R., Bond B., Rice R., Vaughn R.W., McKendrick A., Ayad G., Rockwell M.A., Rocchio R. (1987). Ferritin Content in Human Cancerous and Noncancerous Colonic Tissue. Cancer Investig..

[77] Lee S., Song A., Eo W. (2016). Serum Ferritin as a Prognostic Biomarker for Survival in Relapsed or Refractory Metastatic Colorectal Cancer. J. Cancer.

[78] Sun Y., Yokoi K., Li H., Gao J., Hu L., Liu B., Chen K., Hamilton S.R., Fan D., Sun B. (2011). NGAL Expression Is Elevated in Both Colorectal Adenoma-Carcinoma Sequence and Cancer Progression and Enhances Tumorigenesis in Xenograft Mouse Models. Clin. Cancer Res..

[79] Catalán V., Gómez-Ambrosi J., Rodríguez A., Ramírez B., Silva C., Rotellar F., Hernández-Lizoain J.L., Baixauli J., Valentí V., Pardo F. (2011). Up-regulation of the novel proinflammatory adipokines lipocalin-2, chitinase-3 like-1 and osteopontin as well as angiogenic-related factors in visceral adipose tissue of patients with colon cancer. J. Nutr. Biochem..

[80] Cui C., Cheng X., Yan L., Ding H., Guan X., Zhang W., Tian X., Hao C. (2019). Downregulation of TfR1 promotes progression of colorectal cancer via the JAK/STAT pathway. Cancer Manag. Res..

[81] Zhang X.-F., Zhang Y., Zhang X.-H., Zhou S.-M., Yang G.-G., Wang O.-C., Guo G.-L., Yang G.-Y., Hu X.-Q. (2009). Clinical significance of Neutrophil gelatinase-associated lipocalin(NGAL) expression in primary rectal cancer. BMC Cancer.

[82] Hohaus S., Massini G., Giachelia M., Vannata B., Bozzoli V., Cuccaro A., D’Alo’ F., Larocca L.M., Raymakers R.A.P., Swinkels D.W. (2010). Anemia in Hodgkin’s Lymphoma: The Role of Interleukin-6 and Hepcidin. J. Clin. Oncol..

[83] Eshhar Z., Order S.E., Katz D.H. (1974). Ferritin, a Hodgkin’s disease associated antigen. Proc. Natl. Acad. Sci. USA.

[84] Hann H.W., Lange B., Stahlhut M.W., McGlynn K.A. (1990). Prognostic importance of serum transferrin and ferritin in childhood Hodgkin’s disease. Cancer.

[85] Habeshaw J.A., Lister T.A., Stansfeld A.G., Greaves M.F. (1983). Correlation of transferrin receptor expression with histological class and outcome in non-hodgkin lymphoma. Lancet.

[86] Tisi M.C., Bozzoli V., Giachelia M., Massini G., Ricerca B.M., Maiolo E., D’Alo’ F., Larocca L.M., Piciocchi A., Tjalsma H. (2014). Anemia in diffuse large B-cell non-Hodgkin lymphoma: The role of interleukin-6, hepcidin and erythropoietin. Leuk. Lymphoma.

[87] Basuli D., Tesfay L., Deng Z., Paul B., Yamamoto Y., Ning G., Xian W., McKeon F., Lynch M., Crum C.P. (2017). Iron addiction: A novel therapeutic target in ovarian cancer. Oncogene.

[88] Santin A.D., Zhan F., Bellone S., Palmieri M., Cane S., Bignotti E., Anfossi S., Gokden M., Dunn D., Roman J.J. (2004). Gene expression profiles in primary ovarian serous papillary tumors and normal ovarian epithelium: Identification of candidate molecular markers for ovarian cancer diagnosis and therapy. Int. J. Cancer.

[89] Cho H., Kim J.-H. (2009). Lipocalin 2 Expressions Correlate Significantly With Tumor Differentiation in Epithelial Ovarian Cancer. J. Histochem. Cytochem..

[90] Lim R., Ahmed N., Borregaard N., Riley C., Wafai R., Thompson E.W., Quinn M.A., Rice G.E. (2007). Neutrophil gelatinase-associated lipocalin (NGAL) an early-screening biomarker for ovarian cancer: NGAL is associated with epidermal growth factor-induced epithelio-mesenchymal transition. Int. J. Cancer.

[91] Miyamoto T., Kashima H., Suzuki A., Kikuchi N., Konishi I., Seki N., Shiozawa T. (2011). Laser-captured microdissection-microarray analysis of the genes involved in endometrial carcinogenesis: Stepwise up-regulation of lipocalin2 expression in normal and neoplastic endometria and its functional relevance. Hum. Pathol..

[92] Wong Y.F., Cheung T.H., Lo K.W.K., Yim S.F., Siu N.S.S., Chan S.C.S., Ho T.W.F., Wong K.W.Y., Yu M.Y., Wang V.W. (2007). Identification of molecular markers and signaling pathway in endometrial cancer in Hong Kong Chinese women by genome-wide gene expression profiling. Oncogene.

[93] Ito H., Takagi Y., Ando Y., Kubo A., Hashimoto S., Tsutsui F., Kurihara S. (1980). Serum ferritin levels in patients with cervical cancer. Obs. Gynecol..

[94] Hänninen M.M., Haapasalo J., Haapasalo H., Fleming R.E., Britton R.S., Bacon B.R., Parkkila S. (2009). Expression of iron-related genes in human brain and brain tumors. BMC Neurosci..

[95] Barresi V., Tuccari G., Barresi G. (2010). NGAL immunohistochemical expression in brain primary and metastatic tumors. Clin. Neuropathol..

[96] Schonberg D.L., Miller T.E., Wu Q., Flavahan W.A., Das N.K., Hale J.S., Hubert C.G., Mack S.C., Jarrar A.M., Karl R.T. (2015). Preferential Iron Trafficking Characterizes Glioblastoma Stem-like Cells. Cancer Cell.

[97] Prior R., Reifenberger G., Wechsler W. (1990). Transferrin receptor expression in tumours of the human nervous system: Relation to tumour type, grading and tumour growth fraction. Vichows Arch. A Pathol. Anat..

[98] Han M., Xu R., Wang S., Yang N., Ni S., Zhang Q., Xu Y., Zhang X., Zhang C., Wei Y. (2018). Six-Transmembrane Epithelial Antigen of Prostate 3 Predicts Poor Prognosis and Promotes Glioblastoma Growth and Invasion. Neoplasia.

[99] Calzolari A., Larocca L.M., Deaglio S., Finisguerra V., Boe A., Raggi C., Ricci-Vitani L., Pierconti F., Malavasi F., De Maria R. (2010). Transferrin Receptor 2 Is Frequently and Highly Expressed in Glioblastomas. Transl. Oncol..

[100] Hann H.W., Evans A.E., Siegel S.E., Wong K.Y., Sather H., Dalton A., Hammond D., Seeger R.C. (1985). Prognostic importance of serum ferritin in patients with Stages III and IV neuroblastoma: The Childrens Cancer Study Group experience. Cancer Res..

[101] Koyama S., Fujisawa S., Watanabe R., Itabashi M., Ishibashi D., Ishii Y., Hattori Y., Nakajima Y., Motohashi K., Takasaki H. (2017). Serum ferritin level is a prognostic marker in patients with peripheral T-cell lymphoma. Int. J. Lab. Hematol..

[102] Sharma S., Nemeth E., Chen Y.-H., Goodnough J., Huston A., Roodman G.D., Ganz T., Lichtenstein A. (2008). Involvement of Hepcidin in the Anemia of Multiple Myeloma. Clin. Cancer Res..

[103] Maes K., Nemeth E., Roodman G.D., Huston A., Esteve F., Freytes C., Callander N., Katodritou E., Tussing-Humphreys L., Rivera S. (2010). In anemia of multiple myeloma, hepcidin is induced by increased bone morphogenetic protein 2. Blood.

[104] Gu Z., Wang H., Xia J., Yang Y., Jin Z., Xu H., Shi J., De Domenico I., Tricot G., Zhan F. (2015). Decreased Ferroportin Promotes Myeloma Cell Growth and Osteoclast Differentiation. Cancer Res..

[105] Iannetti A., Pacifico F., Acquaviva R., Lavorgna A., Crescenzi E., Vascotto C., Tell G., Salzano A.M., Scaloni A., Vuttariello E. (2008). The neutrophil gelatinase-associated lipocalin (NGAL), a NF- B-regulated gene, is a survival factor for thyroid neoplastic cells. Proc. Natl. Acad. Sci. USA.

[106] Zhou Q., Chen J., Feng J., Wang J. (2018). E4BP4 promotes thyroid cancer proliferation by modulating iron homeostasis through repression of hepcidin. Cell Death Dis..

[107] Cheng P.-P., Sun Z.-Z., Jiang F., Tang Y.-T., Jiao X.-Y. (2012). Hepcidin expression in patients with acute leukaemia: HEPCIDIN EXPRESSION IN ACUTE LEUKAEMIA. Eur. J. Clin. Investig..

[108] Friedl A., Stoesz S.P., Buckley P., Gould M.N. (1999). Neutrophil Gelatinase-associated Lipocalin in Normal and Neoplastic Human Tissues. Cell Type-specific Pattern of Expression. Histochem. J..

[109] Das Gupta A., Shah V.I. (1990). Correlation of transferrin receptor expression with histologic grade and immunophenotype in chronic lymphocytic leukemia and non-Hodgkin’s lymphoma. Hematol. Pathol..

[110] Khanna V., Karjodkar F., Robbins S., Behl M., Arya S., Tripathi A. (2017). Estimation of serum ferritin level in potentially malignant disorders, oral squamous cell carcinoma, and treated cases of oral squamous cell carcinoma. J. Cancer Res..

[111] Zhang H., Xu L., Xiao D., Xie J., Zeng H., Wang Z., Zhang X., Niu Y., Shen Z., Shen J. (2006). Upregulation of neutrophil gelatinase-associated lipocalin in oesophageal squamous cell carcinoma: Significant correlation with cell differentiation and tumour invasion. J. Clin. Pathol..

[112] Wang H.-J., He X.-J., Ma Y.-Y., Jiang X.-T., Xia Y.-J., Ye Z.-Y., Zhao Z.-S., Tao H.-Q. (2010). Expressions of Neutrophil Gelatinase-Associated Lipocalin in Gastric Cancer: A Potential Biomarker for Prognosis and an Ancillary Diagnostic Test. Anat. Rec..

[113] Cohen C., Shulman G., Budgeon L.R. (1984). Immunohistochemical ferritin in testicular seminoma. Cancer.

[114] Vela D., Vela-Gaxha Z. (2018). Differential regulation of hepcidin in cancer and non-cancer tissues and its clinical implications. Exp. Mol. Med..

[115] Wu X.-N., Su D., Wang L., Yu F.-L. (2014). Roles of the hepcidin–ferroportin axis and iron in cancer. Eur. J. Cancer Prev..

[116] Ganz T., Olbina G., Girelli D., Nemeth E., Westerman M. (2008). Immunoassay for human serum hepcidin. Blood.

[117] Lauta V.M. (2003). A review of the cytokine network in multiple myeloma: Diagnostic, prognostic, and therapeutic implications. Cancer.

[118] Wessling-Resnick M. (2010). Iron Homeostasis and the Inflammatory Response. Annu. Rev. Nutr..

[119] Zhang S., Chen Y., Guo W., Yuan L., Zhang D., Xu Y., Nemeth E., Ganz T., Liu S. (2014). Disordered hepcidin–ferroportin signaling promotes breast cancer growth. Cell. Signal..

[120] Kuang Y., Wang Q. (2019). Iron and lung cancer. Cancer Lett..

[121] Blanchette-Farra N., Kita D., Konstorum A., Tesfay L., Lemler D., Hegde P., Claffey K.P., Torti F.M., Torti S.V. (2018). Contribution of three-dimensional architecture and tumor-associated fibroblasts to hepcidin regulation in breast cancer. Oncogene.

[122] Nemeth E., Preza G.C., Jung C.-L., Kaplan J., Waring A.J., Ganz T. (2006). The N-terminus of hepcidin is essential for its interaction with ferroportin: Structure-function study. Blood.

[123] Guo W., Zhang S., Chen Y., Zhang D., Yuan L., Cong H., Liu S. (2015). An important role of the hepcidin–ferroportin signaling in affecting tumor growth and metastasis. Acta Biochim. Biophys. Sin..

[124] Xue D., Zhou C.-X., Shi Y.-B., Lu H., He X.-Z. (2015). Decreased expression of ferroportin in prostate cancer. Oncol. Lett..

[125] Torti S.V., Torti F.M. (2020). Iron: The cancer connection. Mol. Asp. Med..

[126] Chua A.C., Knuiman M.W., Trinder D., Divitini M.L., Olynyk J.K. (2016). Higher concentrations of serum iron and transferrin saturation but not serum ferritin are associated with cancer outcomes. Am. J. Clin. Nutr..

[127] Cross A.J., Sinha R., Wood R.J., Xue X., Huang W.-Y., Yeager M., Hayes R.B., Gunter M.J. (2011). Iron Homeostasis and Distal Colorectal Adenoma Risk in the Prostate, Lung, Colorectal, and Ovarian Cancer Screening Trial. Cancer Prev. Res..

[128] Fan K., Gao L., Yan X. (2013). Human ferritin for tumor detection and therapy: Human ferritin for tumor detection and therapy. Wires Nanomed. Nanobiotechnol..

[129] Daniels T.R., Bernabeu E., Rodríguez J.A., Patel S., Kozman M., Chiappetta D.A., Holler E., Ljubimova J.Y., Helguera G., Penichet M.L. (2012). The transferrin receptor and the targeted delivery of therapeutic agents against cancer. Biochim. Biophys. Acta (BBA) Gen. Subj..

[130] Wang B., Zhang J., Song F., Tian M., Shi B., Jiang H., Xu W., Wang H., Zhou M., Pan X. (2016). EGFR regulates iron homeostasis to promote cancer growth through redistribution of transferrin receptor 1. Cancer Lett..

[131] Calzolari A., Finisguerra V., Oliviero I., Deaglio S., Mariani G., Malavasi F., Testa U. (2009). Regulation of transferrin receptor 2 in human cancer cell lines. Blood Cells Mol. Dis..

[132] Knekt P., Reunanen A., Takkunen H., Aromaa A., Heliövaara M., Hakuunen T. (1994). Body iron stores and risk of cancer. Int. J. Cancer.

[133] Mainous A.G. (2005). Transferrin Saturation, Dietary Iron Intake, and Risk of Cancer. Ann. Fam. Med..

[134] Singer E., Markó L., Paragas N., Barasch J., Dragun D., Müller D.N., Budde K., Schmidt-Ott K.M. (2013). Neutrophil gelatinase-associated lipocalin: Pathophysiology and clinical applications. Acta Physiol..

[135] Chakraborty S., Kaur S., Guha S., Batra S.K. (2012). The multifaceted roles of neutrophil gelatinase associated lipocalin (NGAL) in inflammation and cancer. Biochim. Biophys. Acta (BBA) Rev. Cancer.

[136] Wu T. (2004). Serum iron, copper and zinc concentrations and risk of cancer mortality in US adults. Ann. Epidemiol..

[137] Tran K.T., Coleman H.G., McCain R.S., Cardwell C.R. (2019). Serum Biomarkers of Iron Status and Risk of Primary Liver Cancer: A Systematic Review and Meta-Analysis. Nutr. Cancer.

[138] Chang V.C., Cotterchio M., Khoo E. (2019). Iron intake, body iron status, and risk of breast cancer: A systematic review and meta-analysis. BMC Cancer.

[139] Weinberg E.D. (1996). The role of iron in cancer. Eur. J. Cancer Prev..

[140] Wen C.P., Lee J.H., Tai Y.P., Wen C., Wu S.B., Tsai M.K., Hsieh D.P., Chiang H.C., Hsiung C.A., Hsu C.Y. (2014). High serum iron is associated with increased cancer risk. Cancer Res..

[141] Quintana Pacheco D.A., Sookthai D., Graf M.E., Schübel R., Johnson T., Katzke V.A., Kaaks R., Kühn T. (2018). Iron status in relation to cancer risk and mortality: Findings from a population-based prospective study: Iron status in relation to cancer risk and mortality. Int. J. Cancer.

[142] Gaur A., Collins H., Wulaningsih W., Holmberg L., Garmo H., Hammar N., Walldius G., Jungner I., Van Hemelrijck M. (2013). Iron metabolism and risk of cancer in the Swedish AMORIS study. Cancer Causes Control.

[143] Gomes I.M., Maia C.J., Santos C.R. (2012). STEAP Proteins: From Structure to Applications in Cancer Therapy. Mol. Cancer Res..

[144] Amson R.B., Nemani M., Roperch J.P., Israeli D., Bougueleret L., Le Gall I., Medhioub M., Linares-Cruz G., Lethrosne F., Pasturaud P. (1996). Isolation of 10 differentially expressed cDNAs in p53-induced apoptosis: Activation of the vertebrate homologue of the drosophila seven in absentia gene. Proc. Natl. Acad. Sci. USA.

[145] Zhang X., Steiner M.S., Rinaldy A., Lu Y. (2001). Apoptosis induction in prostate cancer cells by a novel gene product, pHyde, involves caspase-3. Oncogene.

[146] Isobe T., Baba E., Arita S., Komoda M., Tamura S., Shirakawa T., Ariyama H., Takaishi S., Kusaba H., Ueki T. (2011). Human STEAP3 maintains tumor growth under hypoferric condition. Exp. Cell Res..

[147] Brookes M.J. (2006). Modulation of iron transport proteins in human colorectal carcinogenesis. Gut.

[148] Xue X., Ramakrishnan S.K., Weisz K., Triner D., Xie L., Attili D., Pant A., Győrffy B., Zhan M., Carter-Su C. (2016). Iron Uptake via DMT1 Integrates Cell Cycle with JAK-STAT3 Signaling to Promote Colorectal Tumorigenesis. Cell Metab..

[149] Lemler D.J., Lynch M.L., Tesfay L., Deng Z., Paul B.T., Wang X., Hegde P., Manz D.H., Torti S.V., Torti F.M. (2017). DCYTB is a predictor of outcome in breast cancer that functions via iron-independent mechanisms. Breast Cancer Res..

[150] Jansson M.D., Lund A.H. (2012). MicroRNA and cancer. Mol. Oncol..

[151] Babu K.R., Muckenthaler M.U. (2019). miR-148a regulates expression of the transferrin receptor 1 in hepatocellular carcinoma. Sci. Rep..

[152] Kindrat I., Tryndyak V., de Conti A., Shpyleva S., Mudalige T.K., Kobets T., Erstenyuk A.M., Beland F.A., Pogribny I.P. (2015). MicroRNA-152-mediated dysregulation of hepatic transferrin receptor 1 in liver carcinogenesis. Oncotarget.

[153] Luo L., Yang R., Zhao S., Chen Y., Hong S., Wang K., Wang T., Cheng J., Zhang T., Chen D. (2018). Decreased miR-320 expression is associated with breast cancer progression, cell migration, and invasiveness via targeting Aquaporin 1. Acta Biochim. Biophys. Sin..

[154] Schaar D.G., Medina D.J., Moore D.F., Strair R.K., Ting Y.I. (2009). miR-320 targets transferrin receptor 1 (CD71) and inhibits cell proliferation. Exp. Hematol..

[155] Kong Y., Hu L., Lu K., Wang Y., Xie Y., Gao L., Yang G., Xie B., He W., Chen G. (2019). Ferroportin downregulation promotes cell proliferation by modulating the Nrf2–miR-17-5p axis in multiple myeloma. Cell Death Dis..

[156] Nekhai S., Gordeuk V.R., Anderson G.J., McLaren G.D. (2012). Iron Metabolism in Cancer and Infection. Iron Physiology and Pathophysiology in Humans.

[157] Lui G.Y., Kovacevic Z., Richardson V., Merlot A.M., Kalinowski D.S., Richardson D.R. (2015). Targeting cancer by binding iron: Dissecting cellular signaling pathways. Oncotarget.

[158] Le N. (2002). The role of iron in cell cycle progression and the proliferation of neoplastic cells. Biochim. Biophys. Acta (BBA) Rev. Cancer.

[159] Yamasaki T., Terai S., Sakaida I. (2011). Deferoxamine for Advanced Hepatocellular Carcinoma. N. Engl. J. Med..

[160] Blatt J. (1994). Deferoxamine in children with recurrent neuroblastoma. Anticancer Res..

[161] Kalinowski D.S., Stefani C., Toyokuni S., Ganz T., Anderson G.J., Subramaniam N.V., Trinder D., Olynyk J.K., Chua A., Jansson P.J. (2016). Redox cycling metals: Pedaling their roles in metabolism and their use in the development of novel therapeutics. Biochim. Biophys. Acta (BBA) Mol. Cell Res..

[162] Kunos C.A., Ivy S.P. (2018). Triapine Radiochemotherapy in Advanced Stage Cervical Cancer. Front. Oncol..

[163] Kalinowski D.S., Richardson D.R. (2005). The Evolution of Iron Chelators for the Treatment of Iron Overload Disease and Cancer. Pharm. Rev..

[164] Yu Y., Gutierrez E., Kovacevic Z., Saletta F., Obeidy P., Suryo Rahmanto Y., Richardson D.R. (2012). Iron Chelators for the Treatment of Cancer. CMC.

[165] Wang L., Li X., Mu Y., Lu C., Tang S., Lu K., Qiu X., Wei A., Cheng Y., Wei W. (2019). The iron chelator desferrioxamine synergizes with chemotherapy for cancer treatment. J. Trace Elem. Med. Biol..

[166] Donfrancesco A., De Bernardi B., Carli M., Mancini A., Nigro M., De Sio L., Casale F., Bagnulo S., Helson L., Deb G. (1995). Deferoxamine followed by cyclophosphamide, etoposide, carboplatin, thiotepa, induction regimen in advanced neuroblastoma: Preliminary results. Italian Neuroblastoma Cooperative Group. Eur. J. Cancer.

[167] Shinoda S., Kaino S., Amano S., Harima H., Matsumoto T., Fujisawa K., Takami T., Yamamoto N., Yamasaki T., Sakaida I. (2018). Deferasirox, an oral iron chelator, with gemcitabine synergistically inhibits pancreatic cancer cell growth in vitro and in vivo. Oncotarget.

[168] Corcé V., Gouin S.G., Renaud S., Gaboriau F., Deniaud D. (2016). Recent advances in cancer treatment by iron chelators. Bioorg. Med. Chem. Lett..

[169] Najlaoui F., Pigeon P., Aroui S., Pezet M., Sancey L., Marrakchi N., Rhouma A., Jaouen G., De Waard M., Busser B. (2018). Anticancer properties of lipid and poly(ε-caprolactone) nanocapsules loaded with ferrocenyl-tamoxifen derivatives. J. Pharm. Pharmacol..

[170] Peter S., Aderibigbe B.A. (2019). Ferrocene-Based Compounds with Antimalaria/Anticancer Activity. Molecules.

[171] Mojžišová G., Mojžiš J., Vašková J. (2014). Organometallic iron complexes as potential cancer therapeutics. Acta Biochim. Pol..

[172] Chitambar C.R. (2017). The therapeutic potential of iron-targeting gallium compounds in human disease: From basic research to clinical application. Pharmacol. Res..

[173] Fung E., Nemeth E. (2013). Manipulation of the hepcidin pathway for therapeutic purposes. Haematologica.

[174] Rupaimoole R., Slack F.J. (2017). MicroRNA therapeutics: Towards a new era for the management of cancer and other diseases. Nat. Rev. Drug Discov..

[175] Greene C.M., Varley R.B., Lawless M.W. (2013). MicroRNAs and liver cancer associated with iron overload: Therapeutic targets unravelled. World J. Gastroenterol..

[176] Shpyleva S.I., Tryndyak V.P., Kovalchuk O., Starlard-Davenport A., Chekhun V.F., Beland F.A., Pogribny I.P. (2011). Role of ferritin alterations in human breast cancer cells. Breast Cancer Res. Treat..

[177] Holohan C., Van Schaeybroeck S., Longley D.B., Johnston P.G. (2013). Cancer drug resistance: An evolving paradigm. Nat. Rev. Cancer.

[178] Dixon S.J., Lemberg K.M., Lamprecht M.R., Skouta R., Zaitsev E.M., Gleason C.E., Patel D.N., Bauer A.J., Cantley A.M., Yang W.S. (2012). Ferroptosis: An iron-dependent form of nonapoptotic cell death. Cell.

[179] Jiang L., Kon N., Li T., Wang S.-J., Su T., Hibshoosh H., Baer R., Gu W. (2015). Ferroptosis as a p53-mediated activity during tumour suppression. Nature.

[180] Hassannia B., Vandenabeele P., Vanden Berghe T. (2019). Targeting Ferroptosis to Iron Out Cancer. Cancer Cell.

[181] Wu Y., Zhang S., Gong X., Tam S., Xiao D., Liu S., Tao Y. (2020). The epigenetic regulators and metabolic changes in ferroptosis-associated cancer progression. Mol. Cancer.

[182] Mai T.T., Hamaï A., Hienzsch A., Cañeque T., Müller S., Wicinski J., Cabaud O., Leroy C., David A., Acevedo V. (2017). Salinomycin kills cancer stem cells by sequestering iron in lysosomes. Nat. Chem..

